# Ghrelin O-Acyl Transferase: Bridging Ghrelin and Energy Homeostasis

**DOI:** 10.1155/2011/217957

**Published:** 2011-09-15

**Authors:** Andrew Shlimun, Suraj Unniappan

**Affiliations:** Laboratory of Integrative Neuroendocrinology, Department of Biology, York University, Toronto, ON, Canada M3J 1P3

## Abstract

Ghrelin O-acyl transferase (GOAT) is a recently identified enzyme responsible for the unique *n-acyl* modification of ghrelin, a multifunctional metabolic hormone. GOAT structure and activity appears to be conserved from fish to man. Since the acyl modification is critical for most of the biological actions of ghrelin, especially metabolic functions, GOAT emerged as a very important molecule of interest. The research on GOAT is on the rise, and several important results reiterating its significance have been reported. Notable among these discoveries are the identification of GOAT tissue expression patterns, effects on insulin secretion, blood glucose levels, feeding, body weight, and metabolism. Several attempts have been made to design and test synthetic compounds that can modulate endogenous GOAT, which could turn beneficial in favorably regulating whole body energy homeostasis. This paper will focus to provide an update on recent advances in GOAT research and its broader implications in the regulation of energy balance.

## 1. Introduction

Ghrelin is a gut hormone discovered in 1999 by Dr. Kojima and colleagues in the laboratory of Dr. Kangawa [1]. It is the first known endogenous ligand of the growth hormone secretagogue receptor 1a (GHS-R1a), which is now known as the ghrelin receptor [2]. Since its discovery, ghrelin has been identified and functions characterized in a large number of animals [3, 4]. A unique aspect of ghrelin, the only known orexigenic hormone from the gut, is the presence of a posttranslational acyl modification, mainly the attachment of the octanoyl group to the third serine residue of the N-terminal region of the peptide [3, 4]. Several variations of this modification were found [3, 4], while the mechanisms that result in this modification, which is critical for many biological actions of ghrelin remained elusive. A decade of waiting ended in 2008 when two research teams independently identified the membrane bound O-acyl transferase (MBOAT) responsible for ghrelin acylation and named it the ghrelin o-acyl Transferase (GOAT) [5, 6].

## 2. Discovery and Characterization of GOAT

**Figure 1:**
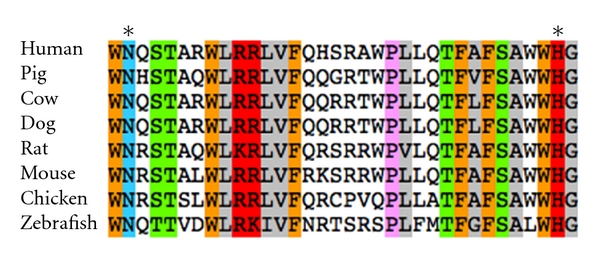
Alignment of amino acid sequences in the highly conserved catalytic regions of GOAT. The online sequence analysis tool available at the following link was used to generate the figure: http://www.protocol-online.org/tools/sms2/color_align_prop.html. The proposed catalytic residues (asparagine and histidine) of GOAT are marked by asterisks. Partial sequences of GOAT were obtained from full-length amino acid sequences obtained from the GenBank. Accession numbers of sequences are human (NP_001094386.1), pig (NP_001177352.1), cow (NP_001179186.1), dog (NP_001188260.1), rat (NP_001100787.2), mouse (NP_001119786.1), chicken (NP_001186218.1), and zebrafish (NP_001116416.1).

Yang et al. [5] first identified 16 MBOAT protein sequences from the mouse genome. They found the 11 putative catalytic regions in these BOATs highly conserved among the 16 sequences, and it all contained the asparagine and histidine residues thought to be involved in the catalysis. Three different murine endocrine cell lines (AtT-20, INS-1, and MIN-6) were transfected with the preproghrelin sequence and were found to produce acylated ghrelin when cotransfected with the GOAT sequence. Mutation of the third serine to alanine prevented the acylation of ghrelin by GOAT, indicating that the third serine is indeed the amino acid to which the moiety is attached. It was also determined that both asparagine in position 307 and histidine in position 338 of mouse GOAT are essential for the catalytic activity of this enzyme (Figure 1). These results provide the first published [5] evidence for GOAT.

**Figure 2:**
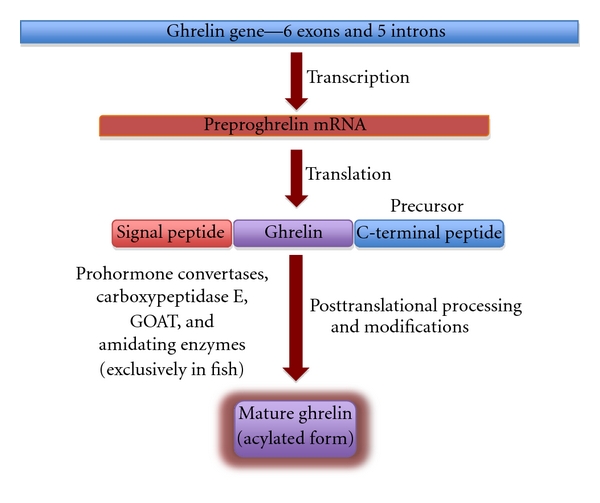
Scheme showing formation of mature, acylated ghrelin from ghrelin gene. Ghrelin gene in humans is comprised of six exons and five introns. Upon transcription, the preproghrelin mRNA is formed, which translates to produce the precursor peptide that contains a signal peptide, mature peptide (ghrelin), and the C-terminal peptide. After posttranslational cleavage and processing by various enzymes including the prohormone convertases, carboxypeptidase E, amidating enzymes, and GOAT, the amidated, acylated mature ghrelin is formed. Amidated ghrelin is currently reported only in fish.

Meanwhile, Gutierrez et al. [6] performed gene-silencing experiments to determine whether a member of the MBOAT family could mediate the acylation of ghrelin in human medullary thyroid carcinoma (TT) cells. They discovered that the silencing of GOAT (MBOAT4), but not other MBOAT sequences, results in the attenuation of ghrelin octanoylation [6]. The GOAT gene has been localized to the 8p12 region of the human chromosome 8. Further experiments by Gutierrez et al. [6] determined that only cotransfection of preproghrelin with GOAT, not with other MBOATs, yielded third serine octanoylated ghrelin in HEK-293 cells. The supplementation of the HEK-293 cell medium with lipids from acetate to tetradecanoic acid resulted in the GOAT enabled modification of ghrelin with fatty acids up to tetradecanoic acid. A very high sequence similarity was found among GOAT proteins from humans to zebrafish [5, 6]. Interestingly, zebrafish, rat, and mouse GOAT were able to acylate human ghrelin [6]. Octanoylated ghrelin was undetectable in the blood of GOAT knockout mice, providing strong confirmatory evidence for the critical role of GOAT in acylating ghrelin. Together, the pioneering research by Yang et al. [5] and Gutierrez et al. [6] led to the discovery of GOAT, the sole mediator of the unique acylation found in ghrelin (Figure 2).

More recent *in vitro *studies by Ohgusu and colleagues [7] found that recombinant GOAT can acylate a short peptide, the N-terminal region of ghrelin consisting of just four amino acids (GSSF). This short peptide is considered as the bioactive core of ghrelin, and it provides further support to the notion that this fragment could elicit many physiological processes regulated by the full-length mature ghrelin. GOAT also has a preference for n-hexanoyl coA compared to n-octanoyl CoA. Yang et al. [8] proposed that preproghrelin is octanoylated in the membranes of endoplasmic reticulum and the acylation occurs after the signal peptide is removed from the preproghrelin. These results provide further support for the four amino acid bioactive core of ghrelin, intracellular location of ghrelin modification, and multiple third serine modifications of ghrelin. 

### 2.1. Tissue Distribution and Regulation of GOAT Expression

The discoverers of GOAT found highest levels of GOAT mRNA expression in the stomach and intestine followed by the testis of mice [5] and the pancreas and stomach of humans [6]. A comprehensive reverse transcription-polymerase chain reaction (RT-PCR) tissue distribution study by Sakata et al. [9] was in agreement with this initial finding. GOAT mRNA expression was detected primarily in the mouse stomach and intestine, while other MBOAT family of enzymes were found in several other tissues in addition to the gastrointestinal tract. This result was further confirmed by double label *in situ* hybridization and immunohistochemistry that found GOAT mRNA expression in ghrelin immunopositive cells and neighboring cells within the gastric mucosa. Together, these results indicate that the primary source of GOAT is gastrointestinal cells that are positive for ghrelin. Stengel et al. [10] for the first time reported colocalization of ghrelin and GOAT immunoreactivity in the stomach mucosa of rats and mice. More GOAT positive ghrelin cells were found in mice (~95%) stomach compared to rats (~56%). The nonghrelin positive GOAT cells in rat gastric mucosa were positive for histidine decarboxylase, which helps in vitamin B6 processing. Western blot analyses found two bands, one at ~50 kDa, the expected size of GOAT protein, and a second one at ~100 kDa, which was considered as a dimer. Western blots of plasma detected GOAT in circulation, and blood levels of GOAT were elevated in mice and rats fasted for 24 hours. Intraperitoneal injections of lipopolysaccharide (LPS) resulted in a significant reduction in plasma acylated ghrelin and caused a corresponding decrease in plasma GOAT levels, while a small increase in gut GOAT was detected [11]. This suggests that ghrelin-GOAT system may be involved in the infection-induced decrease in food intake. While species-specific variations exist in the expression of GOAT in the gut, it is now clear that stomach and intestine are major sources of GOAT. 

In agreement with the original studies, An et al. [12] found abundant expression of GOAT mRNA and protein in the whole pancreas, isolated islets and INS-1 cells. Immunolocalization studies found that GOAT-positive cells are mainly present in the periphery of rat islets and no GOAT-insulin colocalization was found, especially in the beta cell rich core of the islets. González et al. [13], using RT-PCR analysis, reported GOAT mRNA expression in the stomach, pancreas, hypothalamus, ovary, serum, placenta, muscle, heart, and adrenal glands of rats. There were no differences in the expression of GOAT mRNA in the stomach mucosa of male rats at postnatal days 10, 25, and 60 [13]. GOAT expression was also detected in murine cartilage explants, human primary chondrocytes, and in both human and mouse chondrocyte cells lines [14]. The expression of GOAT mRNA in murine chondrogenic cell line ATDC-5 showed a gradual increase as the cells differentiated, with the lowest levels being detected in the early stages of differentiation. LPS significantly reduced GOAT mRNA expression in cultured cartilage cells [14]. GOAT mRNA was detected in the hypothalamus and pituitary of mice [15]. It was also found that GOAT mRNA expression in the cultured primary pituitary cells of mice was increased by acyl ghrelin, leptin, and growth hormone releasing hormone, while somatostatin decreased its expression. No effects were found for neuropeptide Y and des-acyl ghrelin on GOAT mRNA expression [15]. GOAT mRNA expression was found increasing with age in the stomach of rats, while it was found inhibited by a decrease in testosterone [16]. Collectively, from all studies available to date, it appears that the gastroenteropancreatic tissues, which play a major role in postprandial satiety and glucose homeostasis, are the most abundant sources of GOAT in mammals. It is also important to note that several metabolic hormones modulate GOAT expression.

### 2.2. GOAT and Energy Balance

To date, two lines of evidences are available indicating the involvement of GOAT in regulating energy homeostasis. The first set of data is originated from GOAT mRNA expression studies during various metabolic states. The second type of results arises from studies directly testing the effects of GOAT by perturbing the endogenous GOAT. Chronic food deprivation for 21 days resulted in a significant increase in ghrelin mRNA expression in the gastric mucosa of rats and a corresponding increase in GOAT mRNA in the same tissue [13]. Intraperitoneal injections of leptin, a satiety signal that relays the status of long-term energy reserves to the brain, also caused a significant increase in GOAT mRNA expression in 48 fasted rats but not in *ad libitum* fed rats. These results indicate that malnutrition and leptin are two regulatory factors that determine GOAT mRNA expression. In contrast, fasting for 12, 24, or 36 hours caused a significant reduction in stomach GOAT mRNA in mice [17]. No differences in gastric GOAT mRNA expression were found in *ad libitum* fed leptin deficient *ob/ob* mice compared to wild-type controls [17]. Meanwhile, Gahete et al. [15] found that plasma acylated ghrelin levels were increased in fasted mice, and this increase coincided with an elevation in the gut, hypothalamic, and pituitary GOAT mRNA expression. While accepting that discrepancies exist between physiological status of animals and species used, it is interesting that GOAT expression in rodents in general is modulated by the metabolic status.

The importance of GOAT on metabolism was further elucidated using GOAT knockout mice, which lack acyl ghrelin in their circulation. These mice have normal body weight compared to wild-type control mice, but they gained significantly less weight and had reduced fat mass when fed on a high-fat diet for eight weeks [17]. The enrichment of diet with fatty acids resulted in a leaner body mass for the GOAT knockout mice, and this reduction was attributed to increased energy expenditure not alterations in feed intake. No differences in glucose homeostasis were found between the knockout and wild-type mice. Transgenic mice overexpressing human GOAT in the liver, when fed with medium chain fatty acid containing diet, produced more acylated ghrelin and had a transient increase in food intake. Overall, GOAT links the lipid intake into whole body energy balance. Zhao et al. [18] determined the body weight and blood glucose levels in the GOAT knockout mice. While *ad libitum* feeding on regular or high-fat diets showed no difference in the metabolic phenotype, calorie-restricted GOAT knockout mice lost more weight and had low blood glucose levels compared to wild-type controls. The adverse effects of GOAT absence on glucose levels were reversed upon exogenous administration of acylated ghrelin or growth hormone administration. Overall, the outcome of this genetic approach highlights the significance of GOAT in acylating ghrelin and regulating the role of ghrelin on energy balance. It is important to consider the possibility of GOAT modulating peptides other than ghrelin. Therefore, whatever effects resulting from GOAT alterations are possibly not exclusively due to the absence of acylated ghrelin, as other metabolically relevant peptides could be affected due to the absence of GOAT. A new review [19] is now available that compares the effects of ghrelin and GOAT knock-out mice. The readers are encouraged to review it for a further discussion on the above aspect.

Modulating GOAT activity could potentially affect biological actions of ghrelin and this aspect could be targeted to design pharmacological approaches to regulate energy intake and body weight. A successful attempt in this direction was made when Yang et al. [8] discovered that the octanoylated short fragment of ghrelin comprised of N-terminal 5 amino acids (GSSFL) could inhibit GOAT activity. This GOAT inhibitory activity was further enhanced when an amidated short peptide was used (GSSFL-NH_2_). It was also reported that a modified ghrelin fragment with the third serine replaced with an octanoylated diaminopropionic acid could enhance the GOAT inhibitory activity to 45-fold. Such inhibitors could therefore be used to attenuate active ghrelin and its effects on metabolism.

In this regard, other GOAT-specific antagonists have also been designed and functionally characterized [20]. GO-CoA-Tat, a bisubstrate analogue that acts as a GOAT inhibitor, was found to decrease acylated ghrelin production from cells stably transfected with preproghrelin and GOAT. Similarly, GO-CoA-Tat administration significantly reduced acylated ghrelin production *in vivo* in mice fed with medium chain fatty acid rich food. In addition, daily administration of GO-CoA-Tat prevented body weight gain in mice fed on a high-fat diet. GOAT inhibition in pancreatic beta cells using the GO-CoA-Tat caused a significant increase in glucose-stimulated insulin secretion, suggesting an inhibitory role for ghrelin in this process. Similar increases in glucose-induced insulin secretion were also found in mice treated with the GOAT antagonist. The expression of mRNA encoding UCP2, a potent inhibitor of insulin secretion, was suppressed 20-fold in GO-CoA-Tat-treated islets. In agreement with these islet effects, An et al. [21] reported that GOAT mRNA expression in islet beta-like INS-1 cells is inhibited by insulin. This effect of insulin was blocked when cells were treated with wortmannin that inhibits PI3 kinase/Akt pathway. From this result, it is clear that the inhibitory effects of insulin on GOAT are mediated via the insulin receptor signaling pathways. Insulin was also found to reduce GOAT promoter activity *in vitro*. In addition, it was also determined that the mammalian target of rapamycin (mTOR) regulates transcription and translation of GOAT. Inhibition of mTOR signaling using rapamycin significantly increased GOAT mRNA in INS-1 cells in a concentration and time dependent manner. Similarly, intraperitoneal injection of rapamycin that reduced mTOR increased GOAT mRNA expression in the pancreas of C57BL/6 mice. In contrast, leucine, an activator of mTOR, attenuated GOAT mRNA expression in mice pancreas. Overexpressing tuberculosis sclerosis complex 1 and 2 (TSC1 and TSC2), the negative regulators of mTOR action, increased GOAT activity in INS-1 cells. Together, these results provide further evidence for an important role for the endogenous GOAT on glucose homeostasis and insulin secretion, and intracellular mechanisms that mediate these effects.

## 3. Perspectives

Currently, GOAT is the only known enzyme that is capable in acylating ghrelin. It is now clear that GOAT is a critical component in making bioactive ghrelin, and thus mediating the physiological functions of ghrelin. Within a short period since its discovery, enough strong evidence have been collected to support a clear and very important role for GOAT in metabolic physiology. GOAT expression is tissue specific, is modulated by a number of factors, and appears to vary among organisms. It is especially interesting that GOAT is regulated by the metabolic status of animals. Whether GOAT expression is altered in metabolic pathophysiology in humans remain unknown. Another fascinating discovery is the development of GOAT inhibitors and its success in preventing weight gain, stimulating insulin secretion and reducing blood glucose levels. If these findings are translatable, inhibition of endogenous GOAT using specific inhibitors could emerge as potential treatment or preventative strategy for diabetes. Similarly, tweaking GOAT biology may reap rewards in the march towards curbing overweight and obesity. While several interesting results have been obtained to date, GOAT research is still in its infancy, and future studies hold the key to further our understanding of this protein and enhancing its utilization for the benefit of humans.
